# Subjective memory concerns and cognitive aging in Puerto Rican older adults: the moderating role of chronic pain

**DOI:** 10.1093/geroni/igag079

**Published:** 2026-07-06

**Authors:** Tyler R Bell, Sadaf A Milani, Caitlin A Northcutt, Nathalie Gider, Ebenezer Martey, Ross Andel, Michael Crowe

**Affiliations:** Department of Psychiatry, University of California San Diego, La Jolla, California, United States; Department of Epidemiology, University of Texas Medical Branch, Galveston, Texas, United States; Department of Epidemiology & Environmental Health, University of Kentucky, Lexington, Kentucky, United States; Department of Cognitive Science, University of California San Diego, San Diego, California, United States; Department of Psychology, University of Alabama at Birmingham, Birmingham, Alabama, United States; Edson College of Nursing and Health Innovation, Arizona State University, Phoenix, Arizona, United States; Department of Psychology, University of Alabama at Birmingham, Birmingham, Alabama, United States

**Keywords:** Puerto Rico, Cognition, Episodic memory, Hispanic aging

## Abstract

**Background and Objectives:**

Older Puerto Rican adults face disproportionately high dementia rates, and subjective memory concerns (SMCs) are often the earliest sign of cognitive risk. The role of chronic pain in shaping SMC-cognition associations is poorly understood. We evaluated 2 SMC items as predictors of cognition and tested whether chronic pain moderated these associations.

**Research Design and Methods:**

Participants without cognitive impairment (*n *= 575; mean age = 83.90) came from the Puerto Rican Elderly: Health Conditions (PREHCO) study and were reassessed approximately 2 years later (*n *= 433). Chronic pain was defined as pain reported at baseline and in prior waves. SMC items and cognition were modeled in adjusted regression models.

**Results:**

Perceived memory difficulty, but not perceived memory decline, was associated with worse baseline cognitive status (β = −.14, *p* = .003) and episodic memory (β = −.12, *p* = .008), and with greater episodic memory decline (β = −.13, *p* = .013). Chronic pain moderated the associations of perceived memory difficulty with baseline (β = −.21, *p* = .037) and change in episodic memory (β = .39, *p* = .006). Among participants with chronic pain, greater perceived memory difficulty corresponded to poorer concurrent episodic memory (β = −.28, *p* = .003) but was not associated with change in episodic memory (*p* = .393). Among those without chronic pain, perceived memory difficulty was not associated with concurrent episodic memory (*p* = .170) but showed a significant association with subsequent decline (β = −.15, *p* = .005).

**Discussion and Implications:**

Associations of perceived memory difficulty with episodic memory differed by chronic pain status: in the chronic pain group, SMCs tracked concurrent memory performance; in those without pain, they were associated with future episodic memory decline.

Innovation and Translational Significance: Subjective memory concerns do not uniformly signal future cognitive risk—their clinical meaning depends on chronic pain status. Perceived memory difficulty tracked current performance among those with pain and future decline among those without. Accounting for pain status may improve cognitive screening accuracy and reduce dementia-risk misclassification in Puerto Rican older adults.

Hispanic older adults in the United States (US), including Puerto Ricans, are rapidly increasing in number and face disproportionate risks for cognitive decline and dementia compared to non-Hispanic Whites (American Geriatrics Society Ethnogeriatrics Committee, 2016). Recent health system data underscore this disparity: analyses of Veterans Affairs Medical Care records show that the age-adjusted prevalence of Alzheimer’s disease and related dementias (ADRD) is about 45% higher for veterans in Puerto Rico compared to those in the mainland US. Similarly, dementia surveillance data show that 10.6% of Medicare enrollees aged 65+ in Puerto Rico meet the “highly likely” ADRD definition, compared to 7.1% nationally, a difference of 45% the Medicare Dementia DataHub (2024) (NORC, 2024). One potential contributor to these disproportionate risks is the high burden of chronic pain among Puerto Rican older adults, who have substantially higher odds of chronic pain than both non-Hispanic Whites and Mexican Americans (Nahin, 2021). Chronic pain has been linked to increased risk of cognitive decline and dementia, including in Puerto Rican populations (Bell et al., 2022a; Khalid et al., 2020; Milani et al., 2023). Yet little is known about how chronic pain alters the significance of subjective memory concerns (SMCs), which are often among the earliest signs of impending dementia (Jessen et al., 2014). Because SMCs are common in people living with chronic pain and are linked to cognitive difficulties, chronic pain may moderate their associations with cognition, determining whether they signal current difficulties or forecast future decline.

SMCs are defined as self-reported challenges in memory recall and learning, and researchers purport that subjective cognitive difficulties can sometimes be the earliest signs of cognitive decline before changes are detectable by neuropsychological testing (Jessen et al., 2014). Studies indicate that over 80% of people who develop dementia report SMCs up to 12 years prior to diagnosis (Jessen et al., 2020). Furthermore, reviews of studies show that SMC items commonly relate to current cognition and future cognitive decline (Burmester et al., 2016; Pike et al., 2022; Wion et al., 2022). Most of these studies, however, have been focused on largely non-Hispanic white samples (>90%) (Wion et al., 2022). The few studies that have included Hispanic participants suggest that cultural factors and health disparities may alter SMCs’ ability to pick up on age- or ADRD-related cognitive decline (Gupta, 2021; Tomaszewski Farias et al., 2025). This leads to the question of the utility of SMCs in detecting the risk of cognitive decline in Hispanic populations, such as Puerto Rican older adults.

One particular health disparity affecting Puerto Rican adults that may influence how SMCs relate to current cognitive performance and subsequent cognitive decline is chronic pain. While older adults with chronic pain do report greater SMCs, this is often observed in young and middle adulthood—prior to age-related cognitive decline (Montejo et al., 2020; Westoby et al., 2009). This is partly because chronic pain at any age is distracting, producing inattention that may in turn affect abilities such as memory and executive functioning (Baker et al., 2018; Eccleston & Crombez, 1999; Moore et al., 2012). In addition, people with chronic pain often have high levels of depressive symptoms, which explain up to one-third of the variance in SMCs (Castel et al., 2021; McCracken & Iverson, 2001).

Two complementary theoretical frameworks motivate this expectation. The cognitive reserve model proposes that life-course exposures compromising neural integrity, such as gray-matter atrophy and connectivity changes linked to chronic pain (Apkarian et al., 2004; Cruz-Almeida et al., 2019; Rodriguez-Raecke et al., 2009), deplete the structural and functional resources that normally buffer age-related decline (Stern, 2009; Stern et al., 2020). Under this framework, SMCs among individuals with chronic pain may reflect already-compromised reserve and ongoing memory issues rather than the preclinical cognitive decline SMCs are typically thought to signal. In parallel, the cognitive-affective interruption model of pain posits that chronic pain continuously competes for limited attentional resources and interacts with depressive mood, generating memory complaints that are longstanding rather than early indicators of incipient dementia (Eccleston & Crombez, 1999). Together, these frameworks suggest that SMCs should more strongly track current performance in chronic pain populations, while retaining a forecasting role for future decline primarily among individuals without chronic pain.

Our primary goal was to test whether chronic pain moderated the associations of two common but distinct measures of SMCs, perceived memory difficulty and perceived memory decline, with cognition and future cognitive decline among older Puerto Rican adults, leveraging longitudinal data from the Puerto Rican Elderly: Health Conditions (PREHCO) study.

Our study had two specific research aims to meet our goal. For our first aim, we evaluated how SMC items related to cross-sectional and longitudinal measures of cognitive status and episodic memory. This analysis clarifies whether the relationships between SMCs and cognition observed in predominantly non-Hispanic White samples also hold true in this population, rather than assuming generalizability. Given that prior research has consistently demonstrated associations of SMCs with current cognition and subsequent cognitive decline (Burmester et al., 2016; Pike et al., 2022; Wion et al., 2022), our strongest a priori hypothesis was that similar relationships would emerge here.

For our second aim, we evaluated whether chronic pain moderated these associations, assessing whether SMCs held different meanings for individuals with versus without chronic pain. By clarifying how these early risk signals relate to cognition in the context of chronic pain, this research aims to inform early intervention strategies to reduce dementia risk in this vulnerable population. For the second study aim, we considered two alternative hypotheses. First, SMCs may be more strongly related to poorer cognition and faster decline among individuals with chronic pain, reflecting the heightened dementia risk among people with chronic pain (Khalid et al., 2020). Alternatively, SMCs may be more strongly associated with decline in those without chronic pain, since in chronic pain populations SMCs are often longstanding and may reflect attentional disruption or depressive symptoms rather than underlying neurodegenerative processes (Hertzog et al., 2018; Hill et al., 2020; Mogle et al., 2020). By testing these competing hypotheses, this aim will clarify how chronic pain shapes the prognostic meaning of SMCs in late life.

## Method

### Participants

The PREHCO study is a longitudinal population-based investigation of community-dwelling older adults in Puerto Rico (Palloni et al., 2013). Researchers leveraged probability-based methods to representatively sample homes with at least one adult over 60 years of age. A multistage, stratified sample design was implemented, with oversampling of individuals over age 80 and regions with a larger proportion of people of African Caribbean descent. After identification of target households, researchers invited older adults to participate in interviews assessing health, lifestyle, psychosocial, and economic factors. Earlier waves of assessment occurred in this cohort, but for the present analyses we focus on Wave 3 (2021–2022) and Wave 4 (2023–2024), as these were the first assessments to include SMC measures.

Wave 3 served as the analytic baseline (*n *= 845; mean age = 85.0, *SD *= 5.0). We restricted analyses to self-report (non-proxy) participants with available SMC data (*n *= 605). The SMC items were administered only to participants completing the interview themselves; approximately 240 Wave 3 respondents participated by proxy (e.g., because of severe hearing loss, profound cognitive impairment, or other health limitations precluding self-report) and were not administered the SMC items by protocol. We further excluded those who were able to participate but had evidence of cognitive impairment (*n *= 30) to focus on SMCs as a preclinical risk factor for dementia. Evidence of cognitive impairment was defined as a Mini-Mental Cabán (MMC) score below 11 at Wave 3, the validated impairment threshold for this instrument in Spanish-speaking Puerto Rican samples (Sánchez-Ayéndez et al., 2003); inability to complete cognitive testing was indicated by interviewer-documented non-completion of the MMC due to sensory, motor, or behavioral barriers. This yielded a final analytic baseline sample of 575 older adults. Participants completed cognitive testing approximately two years later at Wave 4 (*n *= 433; mean age = 86.4, *SD *= 4.4). Wave 3 and Wave 4, therefore, serve as the baseline and follow-up for our analyses. Sample selection is illustrated in Supplementary Figure 1. Sample sizes for measures of interest at baseline and follow-up are presented in Table 1 and Supplementary Table 2, respectively. Based on pwr.f2.test in R (version 4.5.1), our sample size is 90% powered to detect a Cohen’s *d* effect size of .30 (typically thought to be likely clinically meaningful) (Howard et al., 2011).

**Table 1:** Participant characteristics at the analytic baseline (Wave 3), overall and by chronic pain status (*n *= 575).

Characteristic	Overall (*N *= 575)	No chronic pain (*n *= 406)	Chronic pain (*n *= 169)	*p*
**Age, years, *M* (*SD*)**	83.90 (4.11)	84.20 (4.19)	83.40 (3.87)	.033
**Female, *n* (%)**	356 (61.9)	233 (57.4)	123 (72.8)	<.001
**Education, years, *M* (*SD*)**	9.18 (4.25)	9.34 (4.30)	8.80 (4.12)	.166
**Depressive symptoms (GDS-15), *M* (*SD*)**	2.98 (2.82)	2.45 (2.33)	4.26 (3.43)	<.001
**Perceived memory difficulty (1–5), *M* (*SD*)**	3.20 (1.02)	3.06 (1.06)	3.53 (0.84)	<.001
**Perceived memory decline (1–3), *M* (*SD*)**	2.17 (0.52)	2.13 (0.49)	2.27 (0.56)	.003
**Cognitive status (MMC, 0–20), *M* (*SD*)**	15.50 (2.46)	15.40 (2.45)	15.60 (2.50)	.376
**Episodic memory (TICS-27, 0–20), *M* (*SD*)**	5.34 (2.66)	5.38 (2.64)	5.25 (2.71)	.594

*Note.* GDS-15 = Geriatric Depression Scale, 15-item version; MMC = Mini-Mental Cabán; TICS-27 = Telephone Interview Cognitive Screener, 27-item version. *p*-Values are from independent-samples *t*-tests for continuous variables and χ² tests for categorical variables. Participant characteristics at Wave 4 follow-up are provided in Supplementary Table 2.

### Measures

#### Chronic pain

Chronic pain status was determined using pain data from our analytic baseline assessment (Wave 3) and historical data from prior waves (Waves 1 and 2). Across waves, individuals were first asked about whether they suffered from pain in the body, which they confirmed as physical pain [“*dolor físico*”] on a numerical rating scale. Participants reporting any degree of pain on both items were coded as “1,” and those reporting no pain were coded as “0.” Chronic pain was defined as pain reported at our analytical baseline (Wave 3) as well as during prior waves (Waves 1 and 2). We adopted an epidemiologic probabilistic definition of chronic pain rather than a clinical diagnosis. PREHCO does not assess the components required for a clinical chronic pain classification (continuous pain duration in months, specific anatomical location, or the underlying condition), so we used a multi-wave pain persistence indicator, an operationalization with established precedent in population-based aging research with periodic interview assessment. Such studies have been used to link chronic pain to memory decline and dementia pathology (Bell et al., 2022b, 2024, 2025; Whitlock et al., 2017). Within the present sample, the indicator also showed expected group differences across covariates: participants meeting the criterion reported significantly higher depressive symptoms, higher SMCs, and were predominantly female (Table 1), patterns consistent with established chronic-pain characteristics in older adults.

#### Subjective memory concerns

Two distinct measures of SMCs served as the independent variables in this study: *perceived memory difficulty* and *perceived memory decline* measured at Wave 3 (analytical baseline). These items, adapted from the Health and Retirement Study, capture related but distinct aspects of subjective cognition. Perceived memory difficulty reflects current self-rated memory ability and is asked as “How would you rate your memory at the present time?,” rated from 1-Excellent, 2-Very Good, 3-Good, 4-Fair, and 5-Poor. Perceived memory decline reflects perceived change relative to two years earlier and is asked as “Compared to two years ago, would you say your memory is…,” rated from 1-Better, 2-More or less the same, 3-Worse. Consistent with prior work, the two SMCs were analyzed separately rather than combined, as they show distinct associations: perceived memory difficulty is more closely tied to cognitive performance, whereas perceived memory decline relates more strongly to depressive symptoms (Hertzog et al., 2018; Hill et al., 2020; Mogle et al., 2020). All item response levels were included in the statistical models, but figures present collapsed categories for ease of visualization. Because perceived memory difficulty (five response levels) and perceived memory decline (three response levels) have few response categories, continuous treatment requires an equal-interval assumption between adjacent levels; with limited scale resolution, violations of this assumption are more consequential than for scales with many levels. Response distributions were not severely skewed or clustered (perceived memory difficulty: *M* = 3.20, *SD* = 1.02; perceived memory decline: *M* = 2.17, *SD* = 0.52), so the primary concern is the equal-spacing assumption rather than distributional collapse. We followed the standard convention in the aging literature of modeling self-rated memory items as continuous (e.g., Hertzog et al., 2018; Hill et al., 2020; Mogle et al., 2020), which preserves interpretable single coefficients and treats Likert-style self-ratings as indicators of an underlying continuous construct. We also conducted threshold-liability sensitivity analyses (see Supplementary Material), which impose no equal-interval constraint and show little difference in results.

#### Cognitive status

Cognitive status was assessed using the MMC. The MMC was designed as a dementia screening instrument to improve assessments in Spanish-speaking populations and older adults with lower education. The MMC includes items for orientation, immediate and delayed memory (recall of three words), visual memory (episodic memory, drawing a complex figure after 15 s of exposure), and executive function (clock drawing, abstraction). Scores range from 0 to 20, with higher scores representing better cognitive performance. The MMC instrument showed superior sensitivity/specificity compared to a Spanish-translated version of the Mini-Mental State Examination (MMSE) for dementia in a clinic-based sample in Puerto Rico (Sánchez-Ayéndez et al., 2003). MMC scores are on a scale of 0 to 20, with higher scores representing greater cognition. Cognitive impairment was indicated by scores of <11 on the MMC and used for analytic baseline exclusion at Wave 3 (Sánchez-Ayéndez et al., 2003).

#### Episodic memory

Episodic memory was assessed with a subtest from the Telephone Interview Cognitive Screener - 27 Items (TICS-27). Participants were read a list of 10 common words and asked to immediately recall them after hearing the list, and were again asked to recall them ∼5 min later. The number of correctly recalled words immediately and after a delay was summed to form the episodic memory score (0–20), with higher scores reflecting better performance. The TICS-27 episodic memory subtest has been found to be a reliable index of episodic memory function based on a thorough neuropsychological assessment (Truong et al., 2023). While the TICS-27 has a full score to capture cognitive status, we relied on the MMC due to its validation specifically in this population (Sánchez-Ayéndez et al., 2003). While the MMC does include an episodic memory component, it relies on only three words instead of 10.

#### Covariates

Covariates included baseline age, sex, years of education, and depressive symptoms. Depressive symptoms were assessed using a Spanish-language version of the 15-item Geriatric Depression Scale (GDS). Participants were asked about symptoms experienced in the past week and responded either yes or no. Summed scores provide a total depressive symptom score with acceptable reliability (Cronbach’s *α* = .77) (Friedman et al., 2005) and convergent validity with other depressive symptom measures (Kørner et al., 2006). The GDS-15 includes one item that asks about memory relative to same-age peers (“Do you feel that you have more memory problems than other people your age?”). Because perceived memory difficulty was an independent variable of interest, this GDS-15 item was excluded from the depressive symptoms composite score, following previous work (Hill et al., 2019).

### Statistical analysis

#### Descriptive analyses

Descriptive statistics were computed for baseline and follow-up assessments (means and standard deviations for continuous variables; frequencies and percentages for categorical variables). Additionally, for descriptive purposes, we compared study measures between participants with and without chronic pain.

#### Main analyses

##### Aim 1 analysis

To address whether SMC items are related to baseline cognition, we fitted four multiple linear regression models predicting two cognitive measures: (a) cognitive status and (b) episodic memory. Each model included main effects of the SMC item as well as covariates of baseline age, sex, years of education, and depressive symptoms. The baseline model was as such: Y(Baseline Cognitive Measure)i = β0 + β1(Baseline Age) + β2(Sex) + β3(Years of Education) + β4(Subjective Memory Concern Item) + β5(Depressive Symptoms) + ε. These linear regressions were tested in *R* (version 4.5.1) using the *lm* function.

Next, we assessed whether SMC items related to cognitive change by fitting parallel models predicting change in cognitive status and episodic memory. Change scores were calculated using the *residualization* approach, whereby a model was fit with the cognitive measure at follow-up being predicted by cognition scores at baseline. Predicted values of cognitive scores at follow-up are subtracted from the observed score and divided by the root mean square error. This provides a standardized measure whereby a value of −1 indicates a one standard deviation greater cognitive decline than expected. This approach is less affected by ceiling/floor effects and regression to the mean than a difference score approach (Cronbach & Furby, 1970). In addition to adjusting for age, sex, years of education, and depressive symptoms, these models additionally adjusted for the months from baseline to follow-up assessment. The change model was as such: Y(Change in Cognitive Measure)i = β0 + β1(Baseline Age) + β2(Sex) + β3(Years of Education) + β4(Depressive Symptoms) + β5(Months to Follow-up) + β6(Subjective Memory Concern Item) + ε.

##### Aim 2 analysis

To address whether chronic pain moderates the association of SMC items with cognitive function, we extended the previous models to include the main effect of chronic pain and an interaction term of chronic pain with the SMC item. The baseline model was as such: Y(Outcome)i  =  β0 + β1(Baseline Age) + β2(Sex) + β3(Years of Education) + β4(Depressive Symptoms) + β5(Subjective Memory Concern Item) + β6(Chronic Pain) + β7(Chronic Pain × SMC Item) + ε. The change model was as such: Y(Change in Cognitive Measure)i  =  β0 + β1(Baseline Age) + β2(Sex) + β3(Years of Education) + β4(Depressive Symptoms) + β5(Months to Follow-up) + β6(Subjective Memory Concern Item) + β7(Chronic Pain) + β8(Chronic Pain × SMC Item) + ε. For significant interactions, we conducted simple slope analyses using the *emmeans* package to get the stratified associations between SMC items and measures of cognition and cognitive change for participants with chronic pain and for participants without chronic pain.

Statistical significance was evaluated at a two-tailed α = .05. Models were estimated by ordinary least squares using *lm*() in *R*; observations with missing values on any model variable were excluded model-wise. All code is available at https://github.com/trbellucsd/PREHCO_CP_SMC_Paper, and data can be accessed at the National Archive for Computerized Data on Aging (NACDA; https://www.icpsr.umich.edu/web/NACDA/studies/39091). We examined standard linear-model diagnostics for each primary model, which are discussed and detailed in the Supplementary Material. Briefly, visual inspection of residuals-versus-fitted and Q-Q plots showed no substantive departures from linearity, homoskedasticity, or residual normality. Cook’s distance was uniformly well below 1.0 (maximum across all models = 0.139), and all variance inflation factors were below conventional thresholds (max VIF ≤1.52). Models were sex-stratified as a sensitivity analysis given known sex differences in both chronic pain and cognition. These are discussed briefly in the results but are detailed in the Supplementary Material.

## Results

### Participant characteristics

At baseline (Wave 3; *n *= 575), participants had a mean age of 83.90 years (*SD *= 4.11), and 61.9% were female. Twenty-nine percent met criteria for chronic pain, defined as persistent or recurrent pain reported across multiple study waves. As shown in Table 1, participants with chronic pain were slightly older (*d* = 0.19, *p* = .033) and more likely to be female (odds ratio = 1.50, *p* < .001) than those without pain, but did not differ in years of education (*p* = .166). They reported substantially higher depressive symptoms (*d *= 0.66, *p* < .001) and greater SMCs, including perceived memory difficulty (*d *= 0.47, *p* < .001) and perceived memory decline (*d *= 0.27, *p* = .003). Cognitive status and episodic memory did not differ by chronic pain status (*p*s > .05). Participant characteristics at follow-up (Wave 4) are provided in Supplementary Table 2 and showed the same pattern of differences between groups.

### Aim 1: associations between subjective memory concerns and cognition

We first examined whether perceived memory difficulty and perceived memory decline were associated with cognitive status and episodic memory (Table 2). Across the full sample, greater perceived memory difficulty was associated with lower cognitive status (β = −.14, 95% CI [−.23, −.05], *p* = .003) and worse episodic memory (β = −.12, 95% CI [−.21, −.03], *p* = .008) at baseline. It was also associated with a greater decline in episodic memory over time (β = −.13, 95% CI [−.23, −.03], *p* = .013). The association with change in cognitive status was nonsignificant (β = −.06, 95% CI [−.15, .03], *p* = .210). By contrast, perceived memory decline was not significantly related to baseline cognition or change (*p*s > .19).

**Table 2:** Associations of subjective memory concerns with cognition.

Predictor	Outcome	β	*SE*	*t*	*p*
**Perceived memory difficulty**	Cognitive status (baseline)	−.14	.046	−2.99	.003
	Episodic memory (baseline)	−.12	.045	−2.73	.008
	Cognitive status (change)	−.06	.049	−1.25	.210
	Episodic memory (change)	−.13	.051	−2.50	.013
**Perceived memory decline**	Cognitive status (baseline)	−.02	.046	−.37	.715
	Episodic memory (baseline)	−.03	.045	−.68	.495
	Cognitive status (change)	.05	.049	1.04	.298
	Episodic memory (change)	−.07	.053	−1.31	.191

*Note.* The model adjusts for covariates of age at baseline, years of education, and depressive symptoms at baseline as measured by the Geriatric Depression Scale, 15 item version. Models examining cognitive change additionally adjusted for the months between assessments.

Regarding covariates, at baseline, older age was significantly associated with lower cognitive status (βs range = −.18 to −.22, *p*s < .01) and episodic memory (βs range = −.11 to −.16, *p*s < .05). Fewer years of education were also linked to poorer cognitive status and episodic memory (βs range =.18 to .22, *p*s < .001). Higher depressive symptoms were modestly associated with lower episodic memory performance (βs range = −.10 to −.15, *p*s < .05) but were not significantly related to cognitive status after adjusting for age and education. For cognitive change models, older age predicted greater decline in cognitive status (βs = −.13, *p*s < .05), whereas education and depressive symptoms were not significantly associated with change in either cognitive outcome (*p*s > .10).

### Aim 2: moderation of SMC and cognition by chronic pain

We next tested whether chronic pain moderated associations of SMCs with baseline cognition and cognitive decline (Table 3). Significant moderation effects emerged for episodic memory, but not for cognitive status. Chronic pain moderated the associations of perceived memory difficulty with baseline episodic memory (β = −.21, 95% CI [−.41, −.01], *p* = .037) and episodic memory decline (β = .39, 95% CI [.12, .66], *p* = .006). Chronic pain did not significantly moderate the association of perceived memory decline with baseline cognition or changes in cognition (*p*s > .05).

**Table 3:** Moderation of subjective memory concerns and cognition associations by chronic pain.

Predictor	Outcome	β^a^	*SE*	*t*	*p*
**Chronic pain × perceived memory difficulty**	Cognitive status (baseline)	−.20	.108	−1.88	.060
	Episodic memory (baseline)	−.21	.102	−2.09	.037
	Cognitive status (change)	.16	.100	1.61	.109
	Episodic memory (change)	.39	.139	2.77	.006
**Chronic pain × perceived memory decline**	Cognitive status (baseline)	−.03	.089	−.30	.767
	Episodic memory (baseline)	−.15	.086	−1.71	.087
	Cognitive status (change)	−.01	.082	−.07	.946
	Episodic memory (change)	.02	.078	.30	.765

*Note.* The model adjusts for main effects of chronic pain and subjective memory item, as well as covariates of age at baseline, years of education, and depressive symptoms at baseline as measured by the Geriatric Depression Scale, 15 item version. Models examining cognitive change additionally adjusted for the months between assessments.

a β represents the interaction term of chronic pain by subjective memory concern item, adjusted for the main effects of each term.

**Figure 1:**
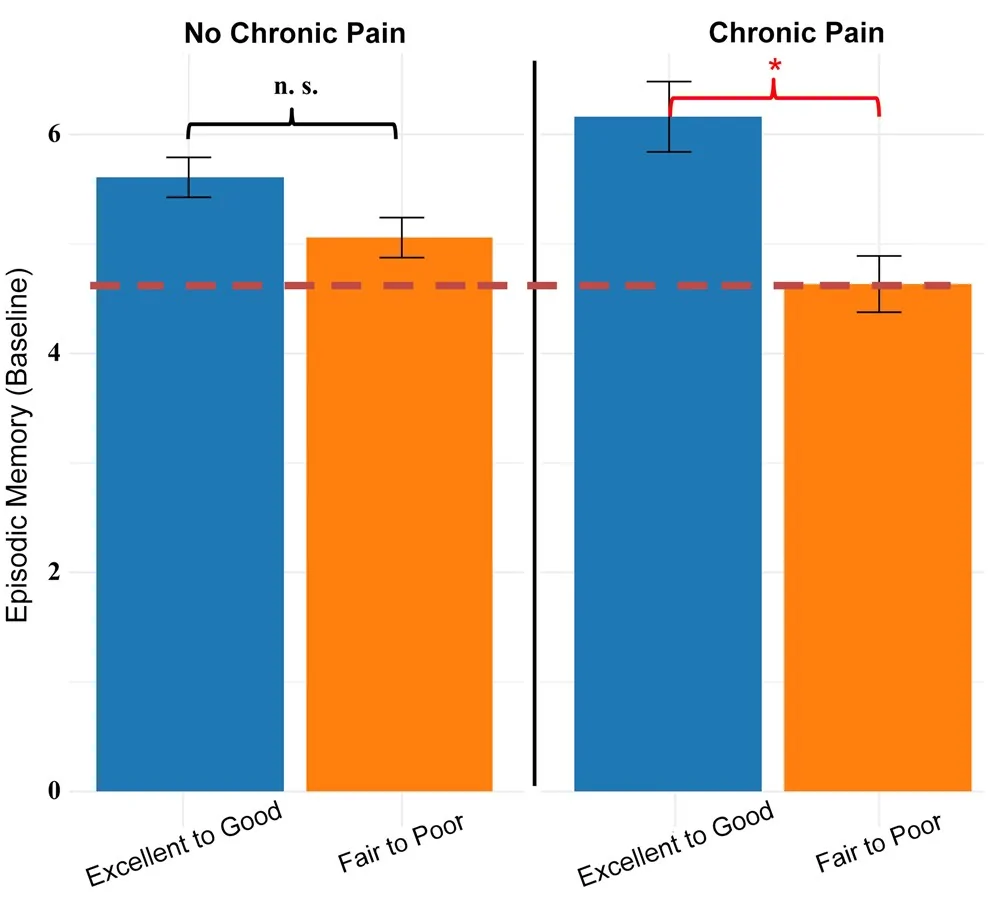
Differences in episodic memory by perceived memory difficulty and chronic pain status. Asterisk (*) indicates differences that are significant at *p* < .05; n.s. indicates nonsignificance at *p* > .05.

**Figure 2:**
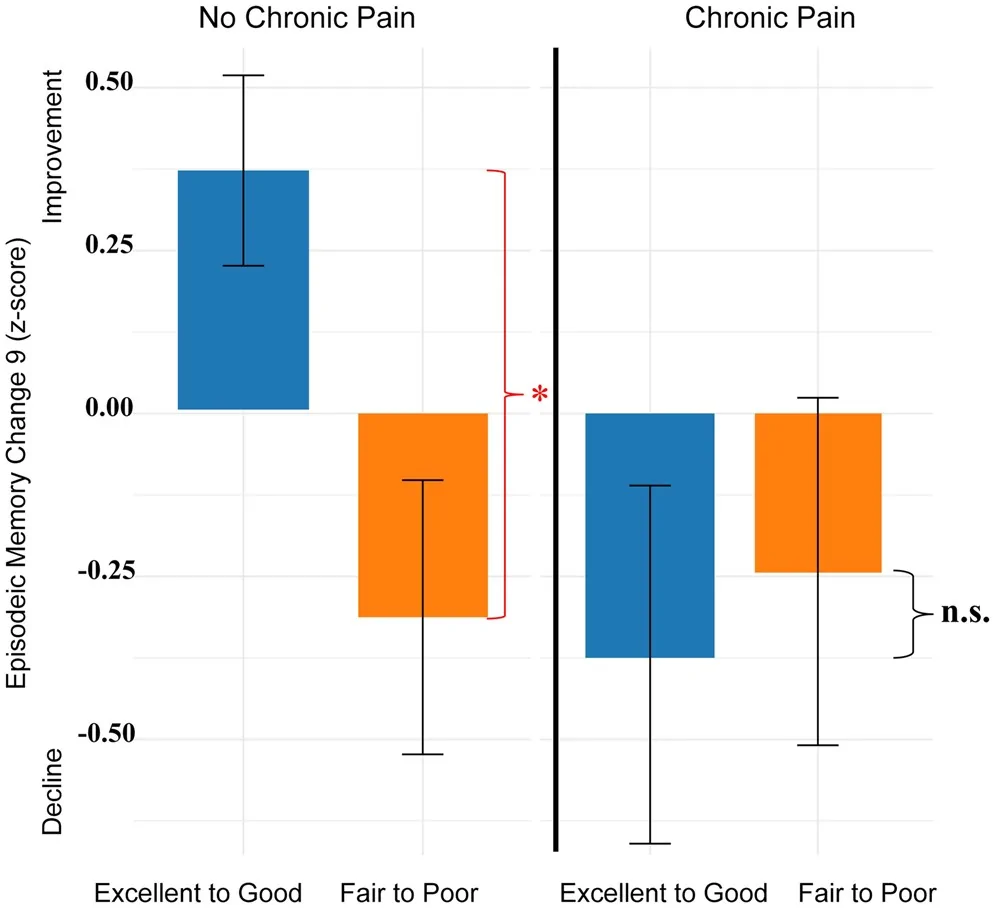
Differences in change in episodic memory by chronic pain status and level of baseline perceived memory difficulty. Asterisk (*) indicates differences that are significant at *p* < .05; n.s. indicates nonsignificance at *p* > .05.

As shown in Table 4, simple slope analyses of significant interactions revealed that among individuals with chronic pain, perceived memory difficulty (β = −.28, 95% CI [−.46, −.10], *p* = .003) was associated with worse baseline episodic memory (see Figure 1) but was not associated with future episodic memory decline (*p* = .393). In contrast, among those without chronic pain, perceived memory difficulty did not relate to baseline episodic memory (*p* = .170) but was significantly associated with greater decline in episodic memory (β = −.15, 95% CI [−.26, −.04], *p* = .005; see Figure 2).

**Table 4:** Simple slope estimates of the associations of subjective memory concerns with cognition by chronic pain status.

Subgroup	Predictor	Outcome	*Β*	*SE*	*p*
**Chronic pain**	Perceived memory difficulty	Episodic memory (baseline)	−.28	.094	.003
**No chronic pain**	Perceived memory difficulty	Episodic memory (baseline)	−.06	.046	.170
**Chronic pain**	Perceived memory difficulty	Episodic memory (change)	.11	.125	.393
**No chronic pain**	Perceived memory difficulty	Episodic memory (change)	−.15	.055	.005

*Note.* The model adjusts for covariates of age at baseline, years of education, and depressive symptoms at baseline as measured by the Geriatric Depression Scale, 15 item version. Models examining cognitive change additionally adjusted for the months between assessments.

### Sensitivity analyses

Sex-stratified estimates of these chronic pain × perceived memory difficulty interactions are reported in Supplementary Table 7. The longitudinal interaction on episodic memory was concentrated among men (β = .47, *p* = .070) with no comparable pattern among women (β = −.02, *p* = .913).

Sensitivity analyses replacing SMC items with threshold-liability scores (Supplementary Tables 3–6) reproduced the primary findings. Aim 1’s main effects were essentially unchanged. The chronic pain × perceived memory difficulty interaction on baseline episodic memory attenuated to non-significance (β = −.12, *p* = .203), whereas the same interaction on episodic memory change remained statistically significant (β = .24, *p* = .049). Stratified simple slopes preserved the same substantive pattern reported in Table 4.

## Discussion

Chronic pain is a recognized risk factor for dementia, yet the earliest signs of cognitive decline in this context remain poorly understood (Khalid et al., 2020). SMCs are often among the first indicators of impairment (Jessen et al., 2014). In people with chronic pain, these concerns are frequently reported earlier in life, reflecting the combined influences of attentional disruption and elevated depressive symptoms (Baker et al., 2018; Castel et al., 2021; Eccleston & Crombez, 1999; Moore et al., 2012). This question is particularly relevant for Puerto Rican older adults, who face disproportionately high rates of both chronic pain and dementia compared to other Latino groups and non-Hispanic Whites (Nahin, 2021; NORC, 2024). Understanding how SMCs relate to baseline cognition and cognitive change in this population, and their interaction with chronic pain, is essential for clarifying whether SMCs function as early markers of decline or instead reflect the burden of pain and mood symptoms. The present findings suggest that associations between SMCs, depressive symptoms, and cognition in older Puerto Ricans with chronic pain may differ from those observed in broader aging populations.

In the full sample, before considering chronic pain, greater perceived memory difficulty was associated with worse overall cognitive status and episodic memory at baseline, as well as greater decline in episodic memory over time. Perceived memory decline, by contrast, was unrelated to cognition at baseline or over time. This divergence reinforces the conceptual distinction between SMC types: perceived memory difficulty, which requires reflection on recent events, likely captures real-world difficulties in learning and recall, whereas perceived memory decline, which relies on more general retrospection, is more vulnerable to factors influencing self-evaluations like negative affectivity (Hertzog et al., 2018; Hill et al., 2020; Mogle et al., 2020).

These findings extend the literature on SMC items and cognition that has been restricted to largely non-Hispanic White samples (>90%) (Burmester et al., 2016; Pike et al., 2022; Wion et al., 2022). Other studies of Latino older adults have found discrepant findings and have largely focused on non-U.S. populations. In Chile, Oyarzún-González et al. (2023) found that the association between perceived memory difficulty and cognitive status was not significant for those without cognitive impairment, but they did not assess episodic memory. In Brazil, Pinho et al. (2023) found that perceived memory difficulty was related to lower episodic memory, but they did not assess cognitive status. Neither study assessed changes in cognition nor the role of perceived memory decline. The present results extend this work to a sample of Puerto Rican older adults, showing that perceived memory difficulty relates cross-sectionally to lower cognitive status and episodic memory and is associated with change in episodic memory, whereas perceived memory decline was not related to cognition or cognitive decline.

The utility of perceived memory decline likely depends on measurement approach: studies using multi-item questionnaires such as the Everyday Cognition Scale and Subjective Cognitive Decline Questionnaire have shown that perceived memory decline relates to episodic memory and is associated with its decline (Márquez et al., 2024; Zlatar et al., 2022). Beyond their improved reliability over single items, multi-item scales focus on specific behaviors rather than a general perception of decline, which is likely more susceptible to affective tone. Although single-item SMC measures remain common because they are brief and easily administered in clinical and epidemiological settings, our findings suggest that such items may lack the sensitivity needed to reliably index cognitive risk in older Puerto Rican adults. Future studies in this population should incorporate multi-item questionnaires that probe specific, everyday memory behaviors (e.g., forgetting appointments, misplacing items, difficulty recalling names), behaviors that prior work links more consistently to objective cognitive performance and future decline, rather than relying on a single global self-rating.

The association of SMC items with cognition at baseline and over time differed by chronic pain status. Among participants with chronic pain, higher perceived memory difficulty was associated with poorer concurrent episodic memory and was not associated with the subsequent two-year change in episodic memory. Among participants without chronic pain, higher perceived memory difficulty was not associated with concurrent episodic memory and was associated with greater subsequent decline in episodic memory. The two groups differ in which temporal aspect of memory their SMCs reflect: perceived memory difficulty aligns with current memory performance in the chronic pain subgroup and with future decline in the no-chronic-pain subgroup. Chronic pain did not moderate associations of SMCs with global cognitive status, nor did it moderate associations of perceived memory decline with any cognitive outcome. The possible mechanisms that might explain these differential patterns, including differences in cognitive reserve, attentional resources, and mood, are considered next.

These differences likely reflect how chronic pain undermines cognitive and neural efficiency before older adulthood. Chronic pain is associated with gray-matter atrophy and disrupted connectivity within medial temporal and default-mode regions that support episodic memory (Apkarian et al., 2004; Rodriguez-Raecke et al., 2009; Vergne-Salle & Bertin, 2021). These early neurobiological alterations may erode passive cognitive reserve, the accumulated structural and functional resources that enable individuals to compensate for age-related neural strain. With reduced reserves, individuals with chronic pain may have less capacity to buffer emerging inefficiencies in memory. Their SMCs may therefore track current memory inefficiency stemming from reduced reserve rather than serve as an early warning of future decline. In contrast, individuals without pain, who maintain greater structural and functional reserve, may follow the more typical trajectory in which SMCs emerge before measurable cognitive decline, reflecting early awareness of memory issues. This interpretation is consistent with evidence that chronic pain accelerates brain-aging processes and limits compensatory adaptation through reduced cognitive reserve (Bell et al., 2022b; Berryman et al., 2014; Buckalew et al., 2008; Cruz-Almeida et al., 2019).

The absence of moderation for cognitive status suggests that the chronic pain × SMC interaction is specific to episodic memory rather than to other domains captured by screeners such as orientation and executive function. However, the analytic exclusion of participants with cognitive impairment at baseline likely restricted the variability of cognitive status in this sample, reducing sensitivity to detect interactions. The relatively short, two-year follow-up period may have further constrained detection of meaningful change in cognitive status, as declines on broad screening measures typically unfold over longer intervals.

As an ancillary finding, in our descriptive analysis, we found that participants with chronic pain had higher ratings of SMCs compared to participants without chronic pain. This pattern is consistent with previous findings in both Hispanic and non-Hispanic populations. For example, in a study of more than 7,000 older adults from the United Kingdom, individuals reporting recent pain had nearly double the likelihood of endorsing memory complaints (Westoby et al., 2009). Similarly, in our prior work, we found that Puerto Rican older adults with chronic pain were more likely to report SMCs than those without pain (Bell et al., 2022a). In that previous study, however, the SMC item was derived from the Geriatric Depression Scale-15, which was beyond the scale’s intended use. The present analyses replicate this association using more commonly used SMC items.

Our study has limitations that warrant consideration. The observed effect sizes were small (βs ∼ .20), which may limit clinical significance for any individual patient. However, small effect sizes are common in cognitive aging research and can still have large public health implications at the population level (Lehert et al., 2015; Livingston et al., 2024). Small effect sizes are also considered a successful change in outcome for interventions designed to reduce cognitive decline (Ball et al., 2002; Swanson et al., 2021). Furthermore, while chronic pain was considered our primary moderator, other prevalent health factors like disease (e.g., cardiovascular disease, diabetes, and depression), sleep health, and medication use may modify associations between SMCs and cognition in this population (Bell et al., 2025; Chen et al., 2019; Gupta, 2021). Examining these factors was beyond the scope of the present study but will be important for future research to delineate distinct and overlapping pathways of cognitive vulnerability.

There are also two notable measurement limitations. First, our chronic pain operationalization was epidemiologic and probabilistic, relying on multi-wave indication of concurrent pain rather than a clinical chronic pain diagnosis requiring information on duration and attributed cause. This means that this chronic pain operationalization may have included some individuals with transient pain, and even in the presence of chronic pain, this was likely to include heterogenous clinical presentations. Future studies in Puerto Rican older adults that incorporate chronic pain diagnoses will allow stronger inferences about the specific pain phenotypes that drive the SMC-cognition associations observed here. Our SMC measures are also limited: they consist of single-item self-ratings rather than validated multi-item instruments (e.g., the Everyday Cognition Scale or the Subjective Cognitive Decline Questionnaire) and are likely less sensitive to subtle or domain-specific cognitive concerns. Comparable single-item SMC measures are used routinely in large population-based aging studies, including the Health and Retirement Study, and the items we used were drawn directly from that tradition; nonetheless, replication using multi-item SMC instruments would strengthen confidence in the associations reported here. Regardless, our study is an important step in assessing the role of SMCs in general levels of cognitive performance and risk of cognitive decline among people with and without chronic pain in a large, representative sample of Puerto Rican older adults.

These findings have several translational implications for clinical monitoring and ADRD risk assessment in Puerto Rican older adults. First, in patients with long-standing chronic pain, memory complaints may correspond to current episodic memory performance rather than signaling impending decline, suggesting value in immediate cognitive screening but not necessarily heightened concern for near-term decline if initial testing is within normal limits. Second, in patients without chronic pain, perceived memory difficulty retained its traditional forecasting role and should be taken seriously as a candidate indicator of preclinical risk warranting closer follow-up. Third, because SMCs in chronic pain populations may partly reflect attentional interruption and depressive symptoms rather than neurodegenerative processes, pairing cognitive screening with pain and depression management may better serve these patients. Fourth, because single global rating items showed limited sensitivity, clinical workflows in this population may benefit from adding brief multi-item, behavior-specific SMC questionnaires (e.g., Everyday Cognition Scale, Subjective Cognitive Decline Questionnaire) to reduce dementia-risk misclassification.

## Conclusion

In Puerto Rican older adults, SMCs were related to worse current levels of cognition, especially episodic memory, though this association was limited to those with chronic pain. Longitudinally, perceived memory difficulty was associated with decline in episodic memory only among participants without pain, suggesting that in chronic pain populations, SMCs may reflect depleted reserve and current difficulties rather than early markers of future decline. By contrast, in those without chronic pain, SMCs retained prognostic value for subsequent decline. These findings highlight the importance of accounting for chronic pain when interpreting SMCs in aging populations and underscore the need for tailored approaches in groups, such as Puerto Rican older adults, who face disproportionate burdens of both pain and dementia.

## Supplementary Material

igag079_Supplementary_Data

## Data Availability

Data from the Puerto Rican Elderly: Health Conditions (PREHCO) study are publicly available at the project website (http://prehco.rcm.upr.edu) and through the Inter-university Consortium for Political and Social Research (ICPSR; https://www.icpsr.umich.edu/web/NACDA/studies/39091). Analysis code and materials used in this article are available at https://github.com/trbellucsd/PREHCO_CP_SMC_Paper. This study was not preregistered.
