# Supplementary material for: Subjective memory concerns and cognitive aging in Puerto Rican older adults: the moderating role of chronic pain
Source: Innov Aging. 2026 Jul 6;10(10):igag079. doi: 10.1093/geroni/igag079 (PMC13587249; doi:10.1093/geroni/igag079)
Supplement: Supplementary file 1 [file igag079_supplementary_data.docx]

***Innovation in Aging* Supplementary Material: Subjective memory concerns and cognitive aging in Puerto Rican older adults: The moderating role of chronic pain.**

**Supplementary Results**

**Supplementary Table 1.** Model diagnostics for primary regression models.

**Supplementary Table 2.** Sample characteristics at follow-up (*N* = 433).

**Supplementary Table 3.** Empirical category proportions, internal probit thresholds, and category-specific liability scores for each subjective memory concern item at the analytic baseline.

**Supplementary Table 4.** Threshold-liability re-estimation of subjective memory concern main-effect models on baseline cognition and cognitive change (mirrors main Table 2).

**Supplementary Table 5.** Threshold-liability re-estimation of chronic pain × subjective memory concern interaction models (mirrors main Table 3).

**Supplementary Table 6.** Threshold-liability re-estimation of stratified simple slopes for chronic pain × perceived memory difficulty interactions on episodic memory (mirrors main Table 4).

**Supplementary Table 7.** Sex-stratified sensitivity analyses of chronic pain moderation models.

**Supplementary Figure 1.** Flowchart of sample selection. Note. SMC = subjective memory concerns.

**Supplementary Results**

**Sensitivity analysis**

To evaluate whether the equal spacing assumption for the ordinal SMC variables affected the results, models were re-run, replacing Likert scores with threshold liabilities. Liability scores were derived in two steps. First, internal probit thresholds for each SMC item were obtained as the inverse standard normal of the empirical cumulative response proportions at baseline (Wave 3). Second, each respondent was assigned the conditional expected value of a standard normal liability given their observed category, E[Z | tk-1 < Z < tk] = (φ(tk-1) - φ(tk)) / (Φ(tk) - Φ(tk-1)). Liability scores were entered in place of the raw ordinal SMC values in the same lm() specifications as the primary analyses (Supplementary Tables 4-6: covariates of baseline age, sex, years of education, and depressive symptoms; change models additionally adjusted for months between assessments). Supplementary Table 4 reproduces main Table 2; Supplementary Table 5 reproduces main Table 3 and reports the chronic pain × SMC interaction term, adjusted for both main effects. Supplementary Table 6 reproduces main Table 4 and reports stratified simple slopes for the chronic pain × perceived memory difficulty interactions on episodic memory at baseline and change, computed via emmeans::emtrends. Coefficients are standardized. Aim 1 main-effect estimates (Supplementary Table 4) were essentially unchanged from the continuous specifications in main Table 2 in both magnitude and statistical-significance status. Aim 2 interaction estimates (Supplementary Table 5) showed somewhat greater attenuation: the chronic pain × perceived memory difficulty interaction on episodic memory at baseline (β = -.21, *p* = .037 in main Table 3) attenuated to β = -.12 (p = .203) under the threshold-liability specification, whereas the same interaction on episodic memory change remained statistically significant (β = .24, *p* = .049, vs. β = .39, *p* = .006 in main Table 3). Stratified simple slopes within chronic pain groups (Supplementary Table 6) preserved the same substantive pattern as main Table 4: the chronic pain group continued to show a stronger concurrent association of perceived memory difficulty with baseline episodic memory than the no-chronic-pain group, and longitudinal episodic memory decline among the no-chronic-pain group remained robustly predicted by perceived memory difficulty. Substantive conclusions for perceived memory decline (no significant association with cognition or moderation by chronic pain) were unchanged across specifications.

**Model fit statistics**

As shown in Supplementary Table 1, models showed VIF ≤ 1.52, well below conventional thresholds. Maximum Cook's D across all models was 0.139, well below Cook's threshold of 1.0. Shapiro-Wilk and Breusch-Pagan tests are reported for transparency; we interpret these in light of the well-known sensitivity of these tests to large samples, and the substantive conclusions rest on the multicollinearity and influence diagnostics together with visual inspection of Q–Q and residuals-versus-fitted plots, which showed no substantive departures from model assumptions.

**Supplementary Table 1.** Diagnostic statistics for the primary and interaction regression models.

| **Model** | **Max VIF** | **Max Cook's D** | **Shapiro-Wilk W (p)** | **Breusch-Pagan p** |
| --- | --- | --- | --- | --- |
| Perceived memory difficulty → MMC (baseline) | 1.11 | 0.026 | 0.990 (< .001) | .477 |
| Perceived memory difficulty → Episodic memory (baseline) | 1.11 | 0.033 | 0.984 (< .001) | .162 |
| Perceived memory difficulty → MMC (change) | 1.11 | 0.064 | 0.973 (< .001) | .023 |
| Perceived memory difficulty → Episodic memory (change) | 1.12 | 0.139 | 0.986 (< .001) | .009 |
| Perceived memory decline → MMC (baseline) | 1.08 | 0.026 | 0.988 (< .001) | .181 |
| Perceived memory decline → Episodic memory (baseline) | 1.08 | 0.035 | 0.984 (< .001) | .128 |
| CP × Perceived memory difficulty → Episodic memory (baseline) | 1.44 | 0.027 | 0.983 (< .001) | .316 |
| CP × Perceived memory difficulty → Episodic memory (change) | 1.52 | 0.108 | 0.986 (< .001) | .016 |

**Supplementary Table 2.** Participant characteristics at follow-up (Wave 4; *N* = 433).

| **Characteristic** | **Overall**  **(*N* = 433)** | **No Chronic Pain**  **(*n* = 311)** | **Chronic Pain (*n* = 122)** | ***p*** |
| --- | --- | --- | --- | --- |
| Age (years), M (SD) | 85.90 (4.04) | 84.10 (4.11) | 83.20 (3.85) | .028 |
| Education (years), M (SD) | 9.24 (4.26) | 9.41 (4.32) | 8.80 (4.07) | .155 |
| Female, n (%) | 267 (61.7) | 178 (57.2) | 89 (73.0) | <.001 |
| Depressive symptoms (GDS-15), M (SD) | 2.92 (2.86) | 2.43 (2.45) | 4.21 (3.43) | <.001 |
| Perceived memory difficulty (1-5), M (SD) | 3.17 (1.01) | 3.05 (1.02) | 3.48 (.92) | <.001 |
| Perceived memory decline (1-3), M (SD) | 2.12 (.51) | 2.07 (.51) | 2.24 (.48) | .002 |
| Cognitive status (MMC, 0-20), M (SD) | 14.60 (3.33) | 14.60 (3.28) | 14.60 (3.46) | .999 |
| Episodic memory (TICS-27, 0-20), M (SD) | 5.35 (2.76) | 5.43 (2.77) | 5.14 (2.72) | .325 |

*Note.* GDS-15 = Geriatric Depression Scale, 15-item version; MMC = Mini-Mental Cabán; TICS-27 = Telephone Interview Cognitive Screener, 27-item version. *p-*values are from independent-samples t-tests for continuous variables and χ² tests for categorical variables.

**Supplementary Table 3.** Empirical category proportions, internal probit thresholds, and category-specific liability scores for each SMC item at the analytic baseline (*n* = 575).

| **SMC item** | **Response level** | ***n*** | **Proportion** | **Probit threshold** | **Liability score** |
| --- | --- | --- | --- | --- | --- |
| *Perceived memory difficulty* | 1 - Excellent | 53 | .092 | (reference) | -1.793 |
|  | 2 - Very good | 73 | .127 | -1.327 | -1.025 |
|  | 3 - Good | 177 | .308 | -0.775 | -0.333 |
|  | 4 - Fair | 249 | .433 | 0.068 | 0.720 |
|  | 5 - Poor | 23 | .040 | 1.751 | 2.154 |
| *Perceived memory decline* | 1 - Better | 36 | .063 | (reference) | -1.967 |
|  | 2 - More or less the same | 406 | .706 | -1.533 | -0.257 |
|  | 3 - Worse | 133 | .231 | 0.735 | 1.317 |

**Supplementary Table 4.** Threshold-liability re-estimation of SMC main-effect models on baseline cognition and cognitive change (mirrors main Table 2).

| **Predictor** | **Outcome** | ***β*** | ***SE*** | ***t*** | ***p*** |
| --- | --- | --- | --- | --- | --- |
| Perceived memory difficulty (liability) | Cognitive Status (Baseline) | -.142 | .043 | -3.32 | .001 |
|  | Episodic Memory (Baseline) | -.110 | .042 | -2.60 | .009 |
|  | Cognitive Status (Change) | -.085 | .050 | -1.72 | .087 |
|  | Episodic Memory (Change) | -.130 | .051 | -2.54 | .012 |
| Perceived memory decline (liability) | Cognitive Status (Baseline) | -.033 | .043 | -0.76 | .445 |
|  | Episodic Memory (Baseline) | -.027 | .042 | -0.65 | .517 |
|  | Cognitive Status (Change) | .053 | .049 | 1.09 | .275 |
|  | Episodic Memory (Change) | -.066 | .053 | -1.25 | .211 |

**Supplementary Table 5.** Threshold-liability re-estimation of chronic pain × SMC interaction models (mirrors main Table 3).

| **Predictor** | **Outcome** | ***β*** | ***SE*** | ***t*** | ***p*** |
| --- | --- | --- | --- | --- | --- |
| Chronic Pain × Perceived memory difficulty (liability) | Cognitive Status (Baseline) | -.091 | .097 | -0.94 | .348 |
|  | Episodic Memory (Baseline) | -.122 | .096 | -1.27 | .203 |
|  | Cognitive Status (Change) | .143 | .113 | 1.26 | .207 |
|  | Episodic Memory (Change) | .237 | .120 | 1.97 | .049 |
| Chronic Pain × Perceived memory decline (liability) | Cognitive Status (Baseline) | .018 | .087 | 0.21 | .837 |
|  | Episodic Memory (Baseline) | -.150 | .086 | -1.76 | .080 |
|  | Cognitive Status (Change) | -.003 | .102 | -0.03 | .980 |
|  | Episodic Memory (Change) | .117 | .113 | 1.04 | .301 |

**Supplementary Table 6.** Threshold-liability re-estimation of stratified simple slopes for chronic pain × perceived memory difficulty interactions on episodic memory (mirrors main Table 4).

| **Subgroup** | **Predictor** | **Outcome** | ***β*** | ***SE*** | ***p*** |
| --- | --- | --- | --- | --- | --- |
| Chronic Pain | Perceived memory difficulty (liability) | Episodic Memory (Baseline) | -.220 | .091 | .016 |
| No Chronic Pain | Perceived memory difficulty (liability) | Episodic Memory (Baseline) | -.090 | .051 | .081 |
| Chronic Pain | Perceived memory difficulty (liability) | Episodic Memory (Change) | .072 | .115 | .532 |
| No Chronic Pain | Perceived memory difficulty (liability) | Episodic Memory (Change) | -.182 | .062 | .004 |

**Supplementary Table 7.** Sex-stratified sensitivity analyses of chronic pain moderation models.

| **Interaction model / outcome** | **Men (n = 210) β [95% CI], *p*** | **Women (n = 365)**  **β [95% CI], *p*** | **Full sample**  **β [95% CI], *p*** |
| --- | --- | --- | --- |
| CP × Perceived memory difficulty → Episodic memory (baseline) | 0.00 [-0.37, 0.38], .980 | -0.19 [-0.43, 0.05], .125 | -0.21 [-0.41, -0.01], .037 |
| CP × Perceived memory difficulty → Episodic memory (change) | 0.47 [-0.04, 0.99], .070 | -0.02 [-0.31, 0.28], .913 | 0.39 [0.12, 0.66], .006 |
| CP × Perceived memory difficulty → Cognitive status (baseline) | 0.14 [-0.24, 0.52], .474 | -0.25 [-0.49, -0.01], .043 | -0.20 [-0.41, 0.01], .060 |
| CP × Perceived memory difficulty → Cognitive status (change) | 0.21 [-0.32, 0.73], .443 | 0.17 [-0.10, 0.44], .206 | 0.16 [-0.04, 0.36], .109 |

*Note.* Standardized interaction-term β coefficients, 95% confidence intervals, and *p*-values.

**Supplementary Figure 1.** Flowchart of sample selection.

Note. SMC = subjective memory concerns. Starting from the Wave 3 PREHCO sample (n = 845; mean age = 85.0), we restricted analyses to self-report (non-proxy) participants with available SMC data (n = 605), then further excluded those with evidence of cognitive impairment (MMC < 11) or inability to complete cognitive testing (n = 30), yielding a final analytic baseline sample of 575. Of these, 433 completed follow-up cognitive testing at Wave 4.
